# Estimation bias and agreement limits between two common self-report methods of habitual sleep duration in epidemiological surveys

**DOI:** 10.1038/s41598-024-53174-1

**Published:** 2024-02-10

**Authors:** Maria Korman, Daria Zarina, Vadim Tkachev, Ilona Merikanto, Bjørn Bjorvatn, Adrijana Koscec Bjelajac, Thomas Penzel, Anne-Marie Landtblom, Christian Benedict, Ngan Yin Chan, Yun Kwok Wing, Yves Dauvilliers, Charles M. Morin, Kentaro Matsui, Michael Nadorff, Courtney J. Bolstad, Frances Chung, Sérgio Mota-Rolim, Luigi De Gennaro, Giuseppe Plazzi, Juliana Yordanova, Brigitte Holzinger, Markku Partinen, Cátia Reis

**Affiliations:** 1 Department of Occupational Therapy, Faculty of Health Sciences, Ariel University, Ariel, Israel; 2 SleepWell Research Program, Faculty of Medicine, University of Helsinki, Helsinki, Finland; 3 Orton Orthopaedics Hospital, Helsinki, Finland; 4 Department of Global Public Health and Primary Care, University of Bergen, Bergen, Norway; 5 Norwegian Competence Center for Sleep Disorders, Haukeland University Hospital, Bergen, Norway; 6 Institute for Medical Research and Occupational Health, Zagreb, Croatia; 7 Sleep Medicine Center, Charité Universitätsmedizin Berlin, Berlin, Germany; 8 Department of Medical Sciences, Neurology, Uppsala University, Uppsala, Sweden; 9 Department of Biomedical and Clinical Sciences, Linköping University, Linköping, Sweden; 10 Department of Pharmaceutical, Uppsala University, Uppsala, Sweden; 11 Li Chiu Kong Family Sleep Assessment Unit, Department of Psychiatry, The Chinese University of Hong Kong, Sha Tin, Hong Kong SAR China; 12 Sleep-Wake Disorders Unit, Department of Neurology, Gui-de-Chauliac Hospital, CHU Montpellier, INSERM Institute of Neurosciences of Montpellier, University of Montpellier, Montpellier, France; 13 Centre de Recherche CERVO/Brain Research Center, École de Psychologie, Université Laval, Quebec, QC Canada; 14 Department of Clinical Laboratory, National Center Hospital, National Center of Neurology and Psychiatry, Kodaia, Japan; 15 Department of Psychology, Mississippi State University, Starkville, MS USA; 16 South Texas Veterans Health Care System, San Antonio, TX USA; 17 Department of Anesthesia and Pain Management, Toronto Western Hospital, University Health Network, University of Toronto, Toronto, ON Canada; 18 Brain Institute, Physiology and Behavior Department and Onofre Lopes University Hospital, Federal University of Rio Grande do Norte, Natal, Brazil; 19 Department of Psychology, Sapienza University of Rome, Roma, Lazio Italy; 20 IRCCS Fondazione Santa Lucia, Rome, Italy; 21 Irccs Istituto Delle Scienze Neurologiche di Bologna, Bologna, Italy; 22 Department of Biomedical, Metabolic and Neural Sciences, University of Modena and Reggio-Emilia, Modena, Italy; 23 Institute of Neurobiology, Bulgarian Academy of Sciences, Sofia, Bulgaria; 24 Institute for Consciousness and Dream Research, Medical University of Vienna, Vienna, Austria; 25 Department of Clinical Neurosciences, University of Helsinki Clinicum Unit, Helsinki, Finland; 26 Helsinki Sleep Clinic, Terveystalo Healthcare Services, Helsinki, Finland; 27 Católica Research Centre for Psychological - Family and Social Welbeing, Universidade Católica Portuguesa, Lisbon, Portugal; 28 Instituto de Medicina Molecular João Lobo Antunes, Universidade de Lisboa, Lisbon, Portugal

**Keywords:** Neuroscience, Health care, Health occupations

## Abstract

Accurate measurement of habitual sleep duration (HSD) is crucial for understanding the relationship between sleep and health. This study aimed to assess the bias and agreement limits between two commonly used short HSD self-report methods, considering sleep quality (SQ) and social jetlag (SJL) as potential predictors of bias. Data from 10,268 participants in the International COVID Sleep Study-II (ICOSS-II) were used. Method-Self and Method-MCTQ were compared. Method-Self involved a single question about average nightly sleep duration (HSD_self_), while Method-MCTQ estimated HSD from reported sleep times on workdays (HSD_MCTQwork_) and free days (HSD_MCTQfree_). Sleep quality was evaluated using a Likert scale and the Insomnia Severity Index (ISI) to explore its influence on estimation bias. HSD_self_ was on average 42.41 ± 67.42 min lower than HSD_MCTQweek_, with an agreement range within ± 133 min. The bias and agreement range between methods increased with poorer SQ. HSD_MCTQwork_ showed less bias and better agreement with HSD_self_ compared to HSD_MCTQfree_. Sleep duration irregularity was − 43.35 ± 78.26 min on average. Subjective sleep quality predicted a significant proportion of variance in HSD_self_ and estimation bias. The two methods showed very poor agreement and a significant systematic bias, both worsening with poorer SQ. Method-MCTQ considered sleep intervals without adjusting for SQ issues such as wakefulness after sleep onset but accounted for sleep irregularity and sleeping in on free days, while Method-Self reflected respondents’ interpretation of their sleep, focusing on their sleep on workdays. Including an SQ-related question in surveys may help bidirectionally adjust the possible bias and enhance the accuracy of sleep-health studies.

## Introduction

Habitual Sleep Duration (HSD) is a widely investigated parameter due to the number of highly reproducible associations to physical and psychological health outcomes^1,2^. It is common to find that health outcomes of interest deteriorate as self-reported HSD deviates from the reference sleep norm interval^3–7^. Choosing the right tools to estimate HSD is challenging in epidemiological sleep research. The best method to self-report HSD is a sleep diary^8^, but it is generally non-applicable in surveys. Majority of the validated (*vis-a-vis* polysomnography (PSG)) sleep questionnairs, that are routinely used in clinical evaluation to reliably distinguish between individuals with and without sleep disorders, are relatively long^9^. To ensure good compliance and high response rates, tools that have minimal number of items are therefore prioritized in epidemiological surveys^10^.

Assessment of HSD in epidemiological surveys can include single questions such as “How many hours do you usually sleep at night?” (e.g., Pittsburgh Sleep Quality Index—PSQI, Self-Assessment of Sleep Survey—SASS)^11,12^, which assumes that adults provide an accurate global and retrospective approximation of their sleep length. Other HSD estimation methods use two questions about sleep onset and offset times to estimate the sleep interval (e.g., Karolinska Sleep Questionnaire—KSQ, Basic Nordic Sleep Questionnaire—BNSQ, Munich Chronotype Questionnaire—MCTQ); these questions are asked separately for work and free days^13–15^. This method estimates sleep timing and crucial sleep metrics like social jetlag (SJL) and irregular sleep^16^. For example, inconsistent sleep timing is an important risk factor for metabolic abnormalities, even more significant than sleep duration^17^.

Various studies found weak-to-moderate correlations between single items of HSD and objectively measured sleep, however the agreement between different methods is poor—ranging between 2.0 and 3.5 h above and below the difference between the means^1,18–22^. Also, sleep diaries and single-question HSDs, displayed either non-significant or weak associations^1^. Self-assessment and time-in-bed duration calculated from habitual bedtime and wake time (rather than sleep onset and offset times), were recently reported to show disagreement with actigraphy-based sleep duration. Specifically, the single question provided a significant underestimate of HSD while the bed-wake interval agreed well with Time-in-Bed (TIB) but overestimated Total Sleep Time (TST)^18^. These biases and disagreements pose a significant challenge in the accurate assessment of contribution of HSD to physical and psychological health in survey research. Further, a recent methodological review showed that the variability in the questions relating to sleep, such as event definitions (e.g., “go to bed” vs. “fall asleep”), context (e.g., “habitual” vs. “work/free days”) and timeframe (“typical night” vs. “recently”) leads to discrepancies in HSD estimation by different self-report methods^23^. Additionally, perceived sleep quality, insomnia symptoms and social schedules are important factors that can affect self-reported HSD^19^, but the extent of these effects have not been systematically quantified in large cohorts.

Sleep quality refers to the subjective experience of sleep, reflecting a number of quantifiable components of physiological sleep, such as depth of sleep (i.e., amount of slow-wave sleep), sleep continuity (i.e., wake after sleep onset, percentage of time awake, and number of awakenings) and additional internal or external factors (i.e., circadian profile, pain, stress)^24^. Poor sleep quality can lead to overestimation or underestimation of sleep duration^25^. A single question of overall sleep quality using a Likert scale is common in both experimental and epidemiological studies, with a verbal scale providing more stable estimation compared to a numerical scale^10,12^. The Insomnia Severity Index (ISI) is sometimes also used as a proxy for sleep quality^26,27^. Social time pressure refers to the demands and constraints of social obligations that may limit the sleep duration^28^. In industrialized societies, people often experience a high social time pressure on workdays, and a large mismatch between internal biological and social times. This mismatch can be quantified by the difference between mid-sleep point on free and workdays and reflects irregularity of sleep timing, called Social Jet Lag (SJL)^29^. Because self-report questions always encompass more than physiological sleep duration alone, evaluating the differences between common self-report methods used to assess HSD in surveys focusing on the potential predictors of the bias is important. The first objective of this study was to evaluate within-subjects estimation bias and the limits of agreement between two short self-report methods used to assess HSD in a large, global, heterogeneous sample of the International Covid Study II (ICOSS-II) project^30^. The second objective of this study was to address the contribution of subjective Sleep Quality and Social Time Pressure to estimate the HSD bias. The contribution of Sleep Quality was validated vis-à-vis Insomnia Severity Index (ISI)—one of the most widely used tools to assess sleep problems in clinical and community samples^27^.

## Results

The sample consisted of 10,268 participants with a mean age of 43.16 ± 16.80 years (Mean ± standard deviation) and 68.3% were female. Demographic descriptive in Table 1.

**Table 1:** Socio-demographic characteristics and sleep measures of the sample. Mean ± SD or frequency (% of group total).

Variables	Sample total n = 10,268
Age, years	43.2 ± 16.8
18–34	3636 (35.4%)
35–39	2771 (27.0%)
50–64	2522 (24.6%)
65–99	1339 (13.0%)
Gender, female	7012 (68.3%)
Country	
Austria	527 (5.1%)
Brazil	197 (1.9%)
Bulgaria	341 (3.3%)
Canada	464 (4.5%)
Croatia	477 (4.6%)
France	305 (3.0%)
Germany	445 (4.3%)
Finland	1181 (11.5%)
Hong Kong	243 (2.4%)
Israel	352 (3.4%)
Italy	786 (7.7%)
Japan	2581 (25.1%)
Norway	491 (4.8%)
Portugal	408 (4.0%)
Sweden	688 (6.7%)
USA	744 (7.2%)
Other	38 (0.4%)
Ethnicity	
White (Caucasian)	6626 (64.9%)
Asian	2713 (26.6%)
African	153 (1.5%)
Hispanic	212 (2.1%)
Other	503 (4.9%)
Marital status	
Single	3351 (32.6%)
Married/relationship	6026 (58.7%)
Divorce/separated	707 (6.9%)
Widowed	179 (1.7%)
Education	
Primary/elementary/lower secondary school	295 (2.9%)
Secondary/high/vocational school	3184 (31.9%)
University/college or above	6512 (65.2%)
Present work	
Student	1903 (18.5%)
Regular day work	5119 (49.9%)
Irregular day work/freelancer/artist/research	989 (9.6%)
Unemployed	356 (3.5%)
Retired	1205 (11.7%)
At home (no salary)	605 (5.9%)
Temporary laid off	91 (0.9%)
Financial burden	
Not at all	4794 (4.8%)
A little/somewhat	3825 (3.8%)
Much/very much	1467 (1.4%)
Body Mass Index (BMI)	25.0 ± 6.3
Insomnia Severity Index (ISI)	8.5 ± 6.1
0–7; no clinical insomnia	5136 (50.3%)
8–14; subthreshold insomnia	3249 (31.8%)
15–21; moderate insomnia	1502 (14.7%)
22–28; severe insomnia	320 (3.1%)
Sleep quality	
Well	2059 (20.1%)
Rather well	2994 (29.2%)
Neither well nor badly	2658 (25.9%)
Rather badly	1958 (19.1%)
Badly	599 (5.8%)
Habitual sleep duration self-report (HSD_self_), min	418.9 ± 77.2
Habitual sleep duration MCTQweek (HSD_MCTQweek_), min	461.4 ± 75.1
Habitual sleep duration MCTQwork (HSD_MCTQwork_), min	449.0 ± 81.1
Habitual sleep duration MCTQfree (HSDMCTQfree), min	492.3 ± 87.7
Social jetlag (SJL), min	56.5 ± 62.2

### Estimation of habitual sleep duration bias and the agreement between methods

**Figure 1:**
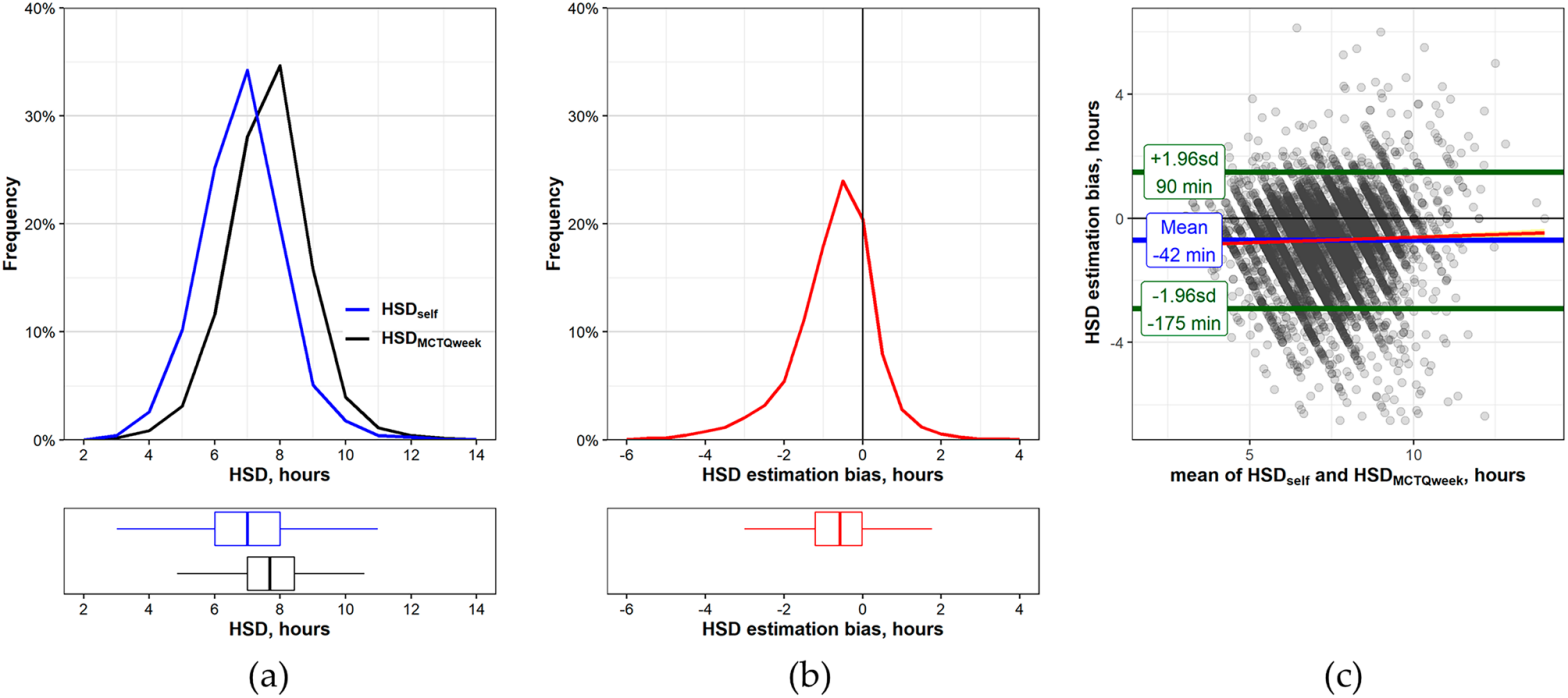
Habitual sleep duration (HSD) by Method-Self and Method-MCTQweek. (**a**) Upper panel—HSD distributions, percent from group total by method: blue line—HSD_self_, black line—HSD_MCTQweek_, 1-h bin. Lower panel—Boxplots of individual HSD by method. Whiskers—max and min values, box borders—75th and 25th percentiles, line through the box—median. (**b**) Upper panel—HSD estimation bias values distribution, percent from group total, 30-min bin. Lower panel—Boxplots of individual HSD estimation bias values. (**c**) Bland–Altman plot comparing Method-Self and Method-MCTQweek. The blue line indicates that the Method-Self sleep duration estimates are on average 42 min shorter than Method-MCTQ estimates. The green lines indicate the 95% limits of agreement (± 1.96SDs). The linear regression line (red) shows that the HSD estimation bias is stable through the whole range values. The two methods only agree to within ± 2.2 h.

Distributions of HSDs from both methods are shown in Fig. 1a, with mean HSD_self_ being shorter (418.9 ± 77.2) than HSD_MCTQweek_ (461.4 ± 75.1). A paired t-test was used to quantify the within-subject difference between methods. A systematic HSD estimation bias was observed (t =  − 63.07, df = 10,267, *p* < 0.001). The mean bias was − 42.41 ± 67.42 min (95% CI of the difference: − 43.72 to − 41.11) and had a normal distribution (Fig. 1b), though HSD_self_ and HSD_MCTQweek_ were significantly positively correlated (rho = 0.604, *p* < 0.001, weighted by age).

The level of agreement between the two HSD assessment methods is visualized using the Bland–Altman plot in Fig. 1c. As neither of the two methods is a “reference”, the bias was compared with the means of the HSD_self_ and the HSD_MCTQweek_ values. To assess whether the bias (represented by the gap between the X axis, and the mean line (blue)) is stable through the whole range of values, a linear regression line (red) was fit to the HSD data points. A Pearson test demonstrated a significant negligible slope (k = 0.034, Beta = 0.02, *p* = 0.03). Finally, the limits of agreement between methods were calculated as: Upper limit $\left[ {\overline{d}\left[ { - 1.96\;{\text{s}}} \right] = - 42.41 - \left( {1.96 \times 67.42} \right) = 175} \right]$; Lower limit $\left[ {\overline{d}\left[ { + 1.96\;{\text{s}}} \right] = - 42.41 + \left( {1.96 \times 67.42} \right) = 90} \right]$. Altogether, the two methods only agreed within ± 133 min, in other words, the HSD_self_ may be 90 min above or 175 min below the HSD_MCTQweek_.

A simple regression model using weighted joint distribution of gender and age by country showed that age was not a significant predictor of the HSD bias (F(1, 10,256) = 2.77, *p* = 0.096, Beta = 0.016). However, women had significantly larger HSD bias than men (t = 4.55, *p* < 0.001, mean difference = 6.6 min), but with a negligibly small effect size (Cohen’s d = 0.097).

### Sleeping well? The HSD estimation bias and the agreement of the methods depend on subjective sleep quality

HSD estimated by both methods negatively correlated with participants’ subjective Sleep Quality, with sleep quality demonstrating a stronger relation to HSD_self_ (Pearson correlations weighted by age: rho =  − 0.334, *p* < 0.01, rho =  − 0.134, *p* < 0.01; HSD_self_ and HSD_MCTQweek_, respectively). Although the two methods are presumably estimating the same construct, using the Fisher r-to-z transformation we found that the two correlation coefficients were also significantly different (z =  − 15.71, *p* < 0.01). The correlation between HSD estimation bias and subjective Sleep Quality was also significant (rho =  − 0.207, *p* < 0.01).

**Figure 2:**
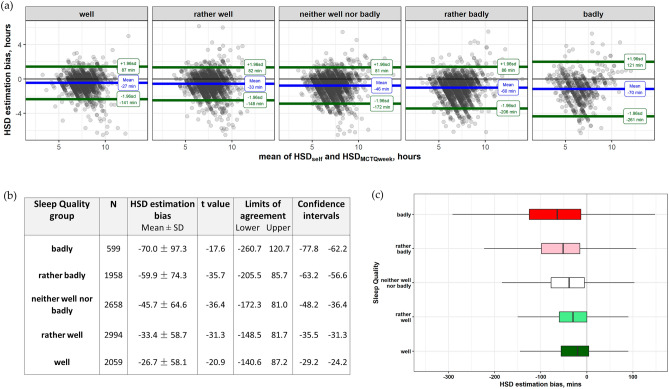
HSD estimation bias by Sleep Quality. (**a**) Bland–Altman plots comparing Method-Self and Method-MCTQweek in five Sleep Quality groups. The blue lines (mean per Sleep Quality group) indicate that underestimation of HSD_self_ relative to HSD_MCTQweek_ increased incrementally as the Sleep Quality worsened: from − 27 min in the “well” sleeping group to − 70 min in the “badly” sleeping group. The 95% limits of agreement (± 1.96 SDs, green lines) also become progressively further apart. (**b**) Statistics of the Bland and Altman plots. (**c**) Boxplots of HSD estimation bias by Sleep Quality. Notations as in Fig. 1c.

To quantify the dependence of the agreement between the two methods in reference to subjective sleep quality, given the large sample size of the ICOSS-II study, HSD bias for each 5 Sleep Quality groups was separately analyzed. One-way ANOVA showed that the estimation bias became more negative as the sleep quality decreased (F(4, 10,256) = 105.16, *p* < 0.001). The results are summarized in Fig. 2. The minimal HDS estimation bias value (− 26.69 ± 58.10 min) and the narrowest range of agreement between methods (± 114 min) were in the group sleeping “well”. The estimation bias and range of agreement became progressively larger with poorer sleep quality. HDS estimation bias in the group sleeping “badly” reached a maximum value of (− 79.97 ± 97.29 min) with a range of agreement of ± 191 min. Post-hoc pairwise comparisons with Bonferroni corrections demonstrated significant distinctions between each of the five sleep quality groups (see supplementary information SI-Table S.1), suggesting underestimation of HSD_self_ relative to HSD_MCTQweek_ increases incrementally.

### Workdays or freedays? The HSD estimation bias and the agreement of methods depends on social time pressure (workdays/free days)

Most participants reported irregular sleep durations across the week. The mean difference between HSD_MCTQwork_ and HSD_MCTQfree_ was − 43.35 ± 78.26 min (449.0 ± 81.1 and 492.3 ± 87.7 min, respectively; paired t-test, t(10,267) =  − 56.13, *p* < 0.001). Accordingly, the distribution of the difference between HSD_MCTQwork_ and HSD_MCTQfree_, with majority of respondents reporting longer sleep duration during free days (percentiles in minutes: 25th = 0, 50th = 30, 75th = 75).

**Figure 3:**
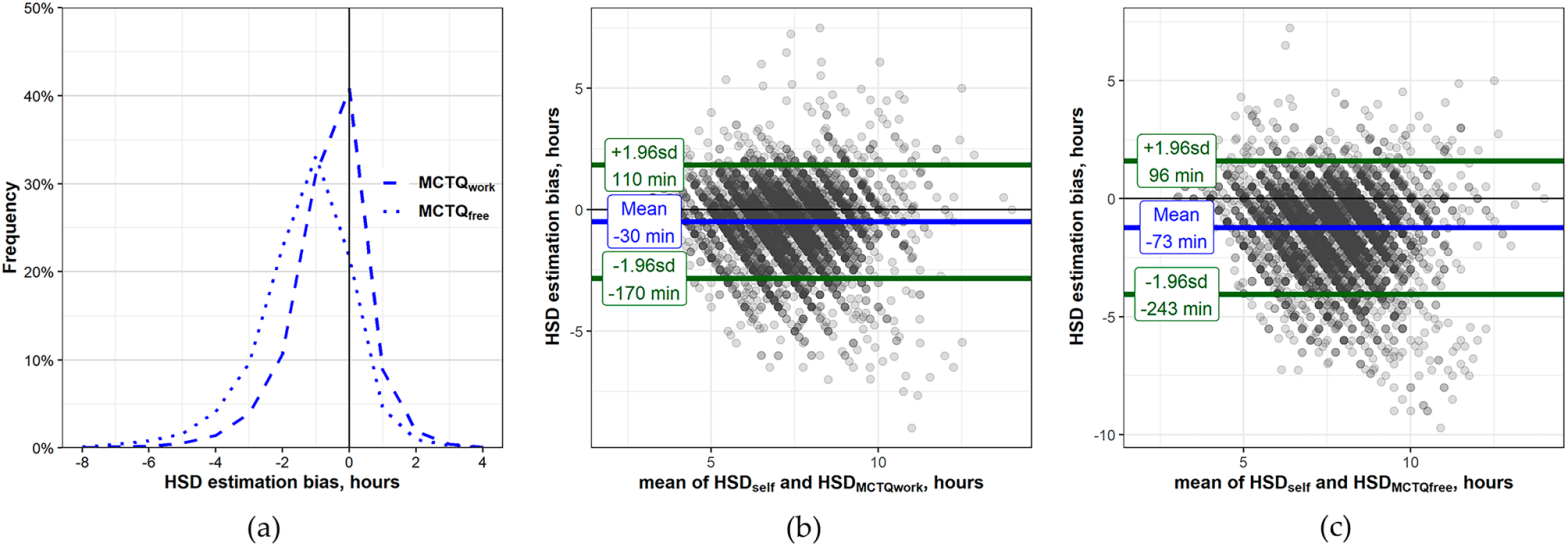
Estimation bias differences between Method-MCTQwork and Method-MCTQfree. (**a**) Habitual sleep duration estimation bias values distribution for workdays and free days, percent from group total. Dotted line—HSD_MCTQfree_, dashed line—HSD_MCTQwork_. (**b**) Bland–Altman plot comparing Method-Self and Method-MCTQwork. Notations as in Fig. 1c. The two methods agree within ± 2.3 h. (**c**) Bland–Altman plot comparing Method-Self and Method-MCTQfree. The two methods agree within ± 2.8 h. Notations as in Fig. 1c.

Next, we tested the hypothesis that HSD_MCTQwork_ would demonstrate a smaller estimation bias and better agreement with HSD_self_ as compared to HSD_MCTQfree_. The mean estimation bias for the HSD_MCTQwork_ was smaller than the HSD_MCTQfree_ (− 30 min, and − 73 min, respectively, Fig. 3a). Further, the agreement limits with the HSD_self_ were similar to the limits of the HSD_MCTQweek_ but better than in HSD_MCTQfree_ (± 140 min vs. ± 169 min, respectively, Fig. 3b,c). The observation that Sleep Quality groups were significantly different from each other was replicated also in HSD_self_–HSD_MCTQwork_ and HSD_self_–HSD_MCTQfree_ comparisons (SI-Tables S.2, S.3)_._

The mean SJL of the sample was 56.5 ± 62.2 min (SJL percentiles, in minutes: 25th = 15, 50th = 45, 75th = 90). There were no significant differences in SJL between the Sleep Quality groups (One-way ANOVA *p* = 0.205).

### The combined contribution of sleep quality and social time pressure on HSD estimation bias

**Figure 4:**
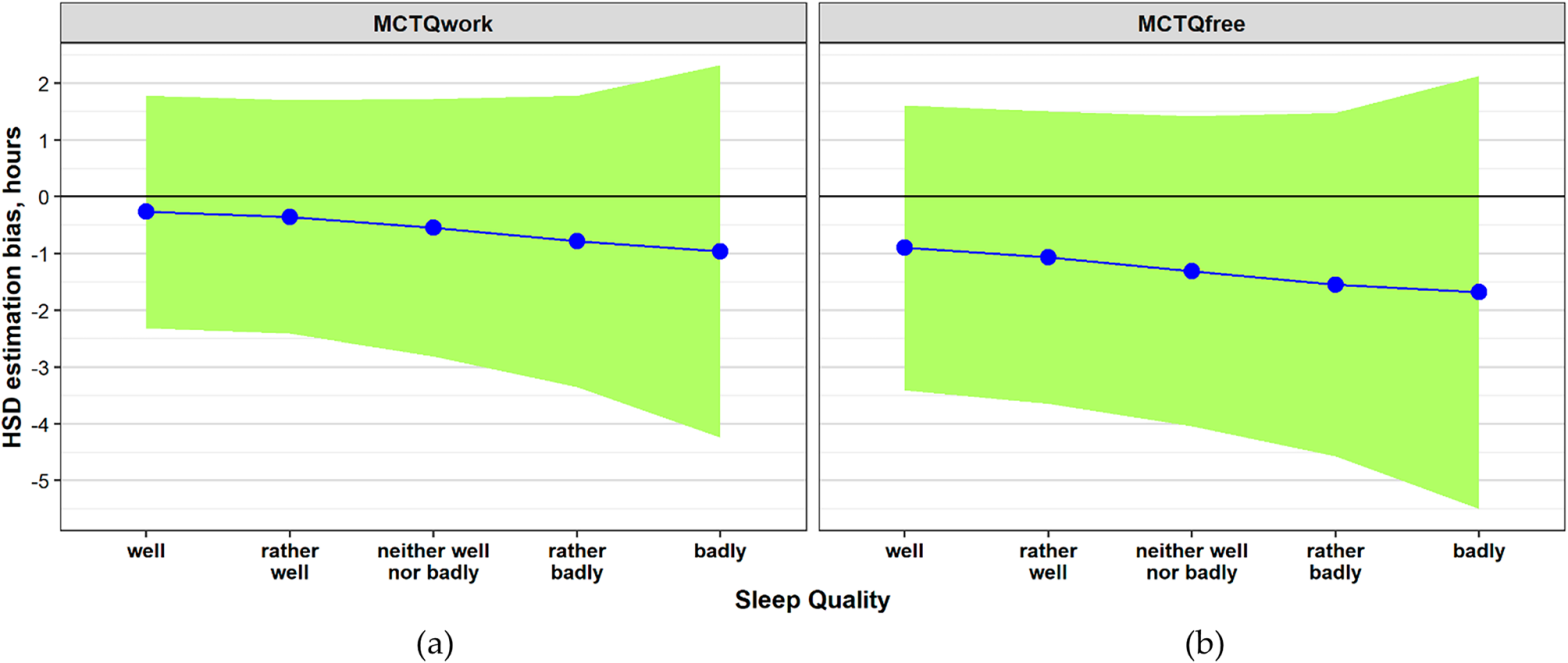
HSD estimation bias as a function of Sleep Quality by (**a**) Method-MCTQwork versus (**b**) Method-MCTQfree. HSD estimation bias values are smaller (closer to zero line) in the Method-MCTQwork as compared with the Method-MCTQfree in all Sleep Quality groups. Green areas around the means—the 95% limits of agreement (± 1.96 SDs). Note that the Method-MCTQwork narrower agreement ranges in all Sleep Quality groups as compared to the Method-MCTQfree.

Having established the effects of Sleep Quality and Social Time Pressure on HSD estimation bias, we presumed that their combination may demonstrate conditions under which the bias is minimal and the agreement between the methods is most reliable. One-way ANOVAs showed that the estimation bias became more negative in both methods as the sleep quality decreased (F(4, 10,263) = 84.312, *p* < 0.001; F(4, 10,263) = 79.65, *p* < 0.001; Method-MCTQ_work_ and Method-MCTQ_free_, respectively). Post-hoc pairwise comparisons with Bonferroni corrections for HSD_MCTQwork_ showed that “well” and “rather well” Sleep Quality groups did not differ, while all other groups showed significant differences (SI-Table S.4). In contrast, for HSD_MCTQfree_, “rather badly” and “badly” Sleep Quality groups were not significantly different from each other, while all other groups showed significant differences (SI-Table S.5). The “well” and “rather well” sleeping groups during workdays showed the best parameters: the mean HSD estimation bias was only − 15.81 ± 62.77 min and the two methods agreed within ± 114 min (Fig. 4a,b).

Weighted least squares stepwise regressions were conducted to examine the extent to which Sleep Quality and Social Time Pressure (represented by SJL) explained the variance in different HSDs and the HSD estimation bias itself. The main model had 5 predictors: Sleep Quality, SJL, age, gender, and BMI. Gender and age by country distribution was used for weighting. The model explained 13.7% of the HSD_self_ variance, 4.2% of the HSD_MCTQweek_ variance, 3.6% of the HSD_MCTQwork_ variance, 10.8% of the HSD_MCTQfree_ variance and 6.9% of the variance in the HSD estimation bias. Leading predictor in all models, except HSD_MCTQfree_, was Sleep Quality, with HSD_self_ demonstrating the largest dependence (12.5% vs. 2.1% vs. 2.1% and 6.2%; HSD_self_, HSD_MCTQweek_ and HSD_MCTQwork_ and HSD estimation bias, respectively). Leading predictor of HSD_MCTQfree_ was SJL (7.4%). Age and gender were significant predictors in most models but explained less than 1% of the variance for all (statistical details in supplementary information SI-Table S.6).

### Comparison between the contributions of sleep quality and ISI score to HSD estimation bias

The contribution of subjective Sleep Quality to the models was assessed using the ISI score, a clinical index of insomnia symptoms severity. Weighted least squares stepwise regressions were re-run with the ISI score used instead of the Sleep Quality and the other four predictors similar to the original model. The variance in HSD_self_, HSD_MCTQweek_ and HSD_MCTQwork_ was primarily explained by the ISI score but the models were less robust (8.4%, 1.4% and 1.5%, respectively, (see details in supplementary information SI-Table S.7). See full statistical details in SI-Table S.7 and SI-Fig. S.1 for the distribution of the HSD estimation bias values by ISI categories. Finally, a model including both Sleep Quality and ISI continuous score as predictors (and SJL, gender, age, and BMI), explained 6.9% of the variance in HSD estimation bias. Note that the ISI score was the least robust contributor accounting only for 0.1% of the variance (SI-Table S.8), demonstrating that ISI score was practically redundant as a predictor of the HSD estimation bias.

## Discussion

It is not clear which self-report method to measure sleep duration can be advised to be used with confidence in large online surveys, since great discrepancies are systematically observed between different methods. Our findings in a large international sample of 10,268 participants also showed poor agreement range (± 133 min), and also indicated systematic and high estimation bias (42.41 ± 67.42 min) between HSD derived from sleep onset and offset and a single question. Thus, for a given person, self-reported sleep duration (HSD_self_) will be almost always lower than self-reported sleep interval (according to HSD_MCTQweek_). For example, if somebody says they sleep 7.5 h a night that means that he/she would estimate their sleep interval as ~ 8h12min (+ 42 min), on average, but the accuracy of this estimation will be very low (± 133 min).

While inaccuracy and problems with face validity of different methods are well recognized in the literature, differences in the dimensionality of the self-report methods, factors that contribute to the poor agreement between them and explain the bias, at least partially, were less studied^18,19,23^. If HSD is systematically under- or overestimated depending on the question, the associations of the health outcomes with sleep duration will also be systematically inflated or flattened^31^. Our findings showed that subjective sleep quality was a strong driver of the estimation bias, the bias almost tripled from the best to worst Sleep Quality group (from 26.69 ± 58.10 to − 79.97 ± 97.29 min). Furthermore, estimation bias changed incrementally with decreasing sleep quality. We also showed that a single question addressing sleep quality contributed to the model explaining the HSD estimation bias more than a multi-item insomnia symptoms severity score. Moreover, having both Sleep Quality and ISI scores as predictors of HSD estimation bias was, in fact, redundant. Sleep quality was also a leading predictor of HSD_self_, HSD_MCTQweek_ and HSD_MCTQwork_, while SJL was a leading predictor of HSD_MCTQfree*.*_ The quantitative estimation of the bias between methods can be used bi-directionally to estimate HSD from one method to the other, if a subjective sleep quality parameter is available.

Our findings therefore indicate that assessing HSD with a single question, or HSD from sleep onset and offset, may capture distinct aspects of sleep duration. The HSD_MCTQweek_ was only subtly influenced by sleep quality, while HSD_self_ and the estimation bias were profoundly sensitive to it. Conversely, the single-question method accounts for poor sleep, but lacks sensitivity to sleep rebound on free days. This may happen because people tend to report the most representative days of the week (i.e., workdays), and lower sleep satisfaction during workdays. This makes the single-question method more susceptible to sleep misperception. Sleep misperception has been found to vary a lot in people from the general population, in patients with insomnia^32^, hypersomnia^33^ and obstructive sleep apnea^34^. These results are in agreement with previous findings, where single questions about sleep duration and sleep quality using the PSQI tool were shown to represent workdays, whereas when the same PSQI questions were asked separately, participants from the general population^35^ had better sleep during free days as well as in clinical populations, and this difference was mediated by SJL^36^. Women had a slightly higher HSD estimation bias compared to men (~ 6 min), and this finding may be explained by the fact that women tend to report lower sleep quality^37^. Interestingly, although sleep duration changes through life^38^, age had no effect on the HSD estimation bias, suggesting that underestimation of HSD_self_ relative to HSD_MCTQweek_ is a stable phenomenon across ages related to sleep quality.

Several limitations exist when interpreting our results. Among those, it was a convenience sample that was collected during COVID-19 pandemic, included unusual participants with a novel health profile of long COVID, and had a clear overrepresentation of women (68.3%). In particular, the data collection period was associated with many changes in the social and personal lives of people across participating countries but note that data was not collected during confinement. Sleep–wake habits during the pandemic were adaptively changing worldwide, with many people working and studying from home^39–41^. Additionally, this study was designed to engage participants who may have had COVID-19 and suffer from symptoms of long COVID^25,30^. Indeed, 9.1% of the sample reported symptoms of long COVID when enrolled in the ICOSS-II study. However, the sensitivity analyses in a sub-group of participants with long-COVID symptoms and in a subgroup of older adults supported the conclusion that HSD bias between methods is a stable trait primarily related to Sleep Quality (see details in the “Methods” and Supplementary Materials sections). Altogether, the web-based survey's generalizability is limited, but maybe partially offset by the large sample size and uniform data acquisition period.

Concerns about self-reported sleep duration accuracy in surveys are longstanding^19,42,43^, even prompting suggestions to exclude it from epidemiological studies^44^. Nevertheless, in large-scale field sleep studies the use of self-report tools is often the only possible option, like in the case of the COVID-19 pandemic^28,30^. Over the last years, many studies showed associations between self-report measures with chronic diseases and mental health^5–7,45^, identifying risk factors, screening for sleep disorders, monitoring changes in the population habits, and understanding the broader public health implications. We believe that researchers using measures of sleep duration based on self-reports should be aware of the meanings and limitations associated with each method, as well as about their disagreement without assuming that all of them reflect physiological sleep to the same extent and strive to add objective measurements of sleep duration or sleep diary when possible.

To conclude, the two methods showed very poor agreement and a significant systematic bias, both worsening with poorer subjective sleep quality. The method using self-reported sleep onset and offset times provides a “raw” calculation of the sleep intervals for work and free days, accounts for irregularities in sleep duration and timing but is inherently insensitive to the frequency and length of awakenings^46,47^. The accuracy of sleep intervals estimations would benefit from inclusion of a wakefulness after sleep onset item, as in Evanger et al.^48^. The single-question sleep duration assessment was found to be associated with sleep quality, and thus may reflect in part how respondents perceive their sleep. However, this method is inherently insensitive to the sleep rebound that occurs on days off^31,49^. We suggest that assessing sleep duration and subjective sleep quality separately for workdays and free days may improve the design of future studies^35,36^. This can be done using either single or two-question approach, in accordance with the specific objectives of the study and, when possible, should include objective measures of sleep. Future studies should evaluate whether including items assessing sleep quality (e.g., single question) and wakefulness after sleep onset may facilitate the implementation of adjustments accounting for potential biases between HSD estimation methods.

## Methods

### Data collection

This study used data from the International Covid Study II (ICOSS-II)^30^, which is an international collaboration between sleep and circadian rhythm experts. Using a web-based anonymous survey, ICOSS-II took place between May to December 2021 in parallel across the following 16 countries using translations to local languages: Austria, Brazil, Bulgaria, Canada, Hong Kong/China, Croatia, Finland, France, Germany, Israel, Italy, Japan, Norway, Portugal, Sweden, USA. The survey used Qualtrics and Redcap platforms. The study conforms to recognized standards by the Declaration of Helsinki. After a brief explanation of the study, the survey was available to participants after obtaining their informed consent to be part of the study. All investigators obtained local ethical committee (REB) approval when applicable (detailed list in supplementary material Table S.8). Due to the anonymous nature of the survey, REB permissions were exempted in some countries.

A total of 16,899 participants opened the link to the ICOSS questionnaire, and 15,859 had valid data. For this study we excluded shift/night workers and subjects reporting severe health conditions (atrial fibrillation, heart failure, stroke, other heart conditions, chronic obstructive pulmonary disease, kidney failure, cancer, immunosuppressive treatment, ongoing Covid-19). For quality control reasons, we excluded participants with HSD < 2.5 h or > 16 h (in either HSD_self_ and HSD_MCTQfree_), with discrepancy in sleep duration estimation of more than 400 min between the two methods, or with missing data in sleep duration and sleep quality parameters. We had a final sample of 10,268 individuals.

### Sleep assessment items and measures

HSD times were assessed twice for each participant using two methods: Method-Self assessment was based on a single-question (i.e., “*How many hours per night you have been sleeping on average CURRENTLY?*”) in the format hh:mm (HSD_self_). The Method-MCTQ used an adapted version of the Munich Chronotype Questionnaire (µMCTQ). The questions were referring to sleep onset and offset timings (reported in 24 h local time format) (i.e., “*At what time do you usually fall asleep at work/free days CURRENTLY?*”, “*At what time do you usually wake up at work/free days CURRENTLY?*”). Separate reports were obtained for workdays and free days, enabling calculation of HSD during workdays and free days (HSD_MCTQwork_, HSD_MCTQfree_) and a weighted weekly average HSD, assuming 5 workdays (HSD_MCTQweek_)^50^. The resolution of the answers was 15 min. Sleep mid-points (between reported sleep onset and offset times) on work- and free days were used to calculate SJL (absolute difference between sleep mid-points on free and workdays)^29^.

Subjective Sleep Quality was reported by participants on a 5-point Likert scale (i.e., well, rather well, neither well or badly, rather badly and badly) as in the BNSQ, in response to the question “How well have you been sleeping CURRENTLY?”. We used these categories to stratify the sample by Sleep Quality groups. Symptoms of insomnia were assessed using the Insomnia Severity Index (ISI), a 7-item questionnaire assessing the nature, severity, and impact of insomnia during “the last month”. A 5-point Likert scale is used to rate each item (0 = no problem to 4 = very severe problem), which provided a total score ranging from 0 to 28. The total score was interpreted as follows: absence of insomnia (0–7); sub-threshold insomnia (8–14); moderate insomnia (15–21); and severe insomnia (22–28)^27^.

### Statistical analysis

Data are reported as mean ± SD or frequency (% of group total). The agreement between the two methods for assessment of HSD (Method-Self and Method-MCTQ) was analyzed using the approach proposed by Bland and Altman^51^. Mean differences between the methods [HSD_self_–HSD_MCTQweek_], or [HSD_self_–HSD_MCTQwork_], or [HSD_self_–HSD_MCTQfree_] were valued as a measure of systematic bias using paired t-tests. The upper and lower limits of agreement were defined as mean difference ± 1.96 × standard deviation with corresponding 95% confidence interval (95% CI). The difference between limits of agreement represents the range of HSD values covering the agreement between the two methods for ~ 95% of the individuals as a measure of precision. Sleep Quality groups were compared using Mann–Whitney or t-tests for continuous variables, according to the type and variables distribution. A simple regression model with weighted joint distribution of gender and age by country was used to estimate the contribution of these demographics to the HSD bias. Multiple regressions were run to evaluate the extent to which Sleep Quality and social time pressure (given by SJL) explained the variance in different HSDs and the HSD estimation bias itself**.** The main model included a set of 5 predictors: Sleep Quality, SJL, and potential demographic confounders previously linked to HSD—including age, gender, and Body Mass Index (BMI). In the validation analysis, ISI score was also used as a predictor. Collinearity tests showed no multicollinearity concerns with the predictors.

The sensitivity analyses to explore potential plausible biases were performed in a sub-group of participants with long-COVID symptoms (SI-Table S.8) and in a subgroup of older adults (> 65 years old, majority after retirement, SI-Table S.9): (1) As the ICOSS-II data were collected 15–21 months after the onset of the COVID-19 pandemic, the first subgroup for sensitivity analysis included 934 (9.1% from total) individuals who met the WHO criteria for long COVID-19^52^. COVID-19 is a recent disorder that impacts sleep and may change the perception of sleep duration with the two estimates. We performed a sensitivity analysis focusing on the HSD estimation and agreement between Method-Self and Method-MCTQ to investigate potential bias in a sub-sample of participants with symptoms of long COVID. (2) Since age and retirement play a major role in sleep habits, sleep quality and social time pressure, the second subgroup for sensitivity analysis included 1187 participants (11.5% from total). The mean age of this group was 71.22 ± 3.68 years old. The data were analyzed using SPSS 29.0 (IBM Corp., Armonk, NY, USA) and R (version 4.0.5).

### Supplementary Information

Supplementary Information.

## Data Availability

We included all the data needed for the evaluation of the conclusions in the “Results” section or in the Supplementary Information file. Additional data related to this article may be requested from the authors.
