# Supplementary material for: Estimation bias and agreement limits between two common self-report methods of habitual sleep duration in epidemiological surveys
Source: Sci Rep. 2024 Feb 10;14:3420. doi: 10.1038/s41598-024-53174-1 (PMC10858912; doi:10.1038/s41598-024-53174-1)
Supplement: Supplementary file 1 — Supplementary Information. [file 41598_2024_53174_MOESM1_ESM.docx]

**Supplementary Materials**

| **SI-Table S.1 HSD estimation bias by Sleep Quality groups: Method-Self** vs. **Method-MCTQweek**.  The estimation bias between method Method-Self and. Method-MCTQweek became progressively larger with poorer sleep quality. The table presents post-hoc pairwise comparisons with Bonferroni corrections and shows that all five Sleep Quality groups were significantly different from each other, suggesting that underestimation of **HSD_self_** relative to **HSD_MCTQweek_** increases incrementally. * The mean difference is significant at the 0.05 level. | | | | | | |
| --- | --- | --- | --- | --- | --- | --- |
| **Sleep Quality groups** | **Sleep Quality groups** | **Estimation bias** | **Std. Error** | **Sig.** | **95% Confidence Interval** | |
|  |  |  |  |  | **Lower Bound** | **Upper Bound** |
| **well**  **N=2059** | **rather well** | 6.67475^*^ | 1.89238 | .004 | 1.3616 | 11.9879 |
|  | **neither well nor badly** | 18.97271^*^ | 1.94050 | <.001 | 13.5245 | 24.4209 |
|  | **rather badly** | 33.24114^*^ | 2.08643 | <.001 | 27.3832 | 39.0991 |
|  | **badly** | 43.27694^*^ | 3.06848 | <.001 | 34.6617 | 51.8921 |
| **rather well**  **N=2994** | **well** | -6.67475^*^ | 1.89238 | .004 | -11.9879 | -1.3616 |
|  | **neither well nor badly** | 12.29795^*^ | 1.76151 | <.001 | 7.3523 | 17.2436 |
|  | **rather badly** | 26.56639^*^ | 1.92108 | <.001 | 21.1727 | 31.9601 |
|  | **badly** | 36.60218^*^ | 2.95854 | <.001 | 28.2957 | 44.9087 |
| **neither well nor badly**  **N=2658** | **well** | -18.97271^*^ | 1.94050 | <.001 | -24.4209 | -13.5245 |
|  | **rather well** | -12.29795^*^ | 1.76151 | <.001 | -17.2436 | -7.3523 |
|  | **rather badly** | 14.26843^*^ | 1.96850 | <.001 | 8.7416 | 19.7953 |
|  | **badly** | 24.30423^*^ | 2.98955 | <.001 | 15.9107 | 32.6978 |
| **rather badly**  **N=1958** | **well** | -33.24114^*^ | 2.08643 | <.001 | -39.0991 | -27.3832 |
|  | **rather well** | -26.56639^*^ | 1.92108 | <.001 | -31.9601 | -21.1727 |
|  | **neither well nor badly** | -14.26843^*^ | 1.96850 | <.001 | -19.7953 | -8.7416 |
|  | **badly** | 10.03580^*^ | 3.08626 | .012 | 1.3707 | 18.7009 |
| **badly**  **N=599** | **well** | -43.27694^*^ | 3.06848 | <.001 | -51.8921 | -34.6617 |
|  | **rather well** | -36.60218^*^ | 2.95854 | <.001 | -44.9087 | -28.2957 |
|  | **neither well nor badly** | -24.30423^*^ | 2.98955 | <.001 | -32.6978 | -15.9107 |
|  | **rather badly** | -10.03580^*^ | 3.08626 | .012 | -18.7009 | -1.3707 |
|  | | | | | | |
| **Table S-2 HSD estimation bias by Sleep Quality groups: Method-Self** vs. **Method-MCTQwork**.  The estimation bias between method Method-Self and. Method-MCTQwork also became progressively larger with poorer sleep quality. The table presents post-hoc pairwise comparisons with Bonferroni corrections and shows that all five Sleep Quality groups were significantly different from each other, suggesting that underestimation of **HSD_self_** relative to **HSD_MCTQweek_** increases incrementally. * The mean difference is significant at the 0.05 level. | | | | | | |
| **Sleep Quality groups** | **Sleep Quality groups** | **Estimation bias** | **Std. Error** | **Sig.** | **95% Confidence Interval** | |
|  |  |  |  |  | **Lower Bound** | **Upper Bound** |
| **well**  **N=2059** | **rather well** | 5.29899 | 2.01765 | .086 | -.3658 | 10.9638 |
|  | **neither well nor badly** | 16.73277^*^ | 2.06896 | <.001 | 10.9239 | 22.5417 |
|  | **rather badly** | 30.97210^*^ | 2.22454 | <.001 | 24.7264 | 37.2178 |
|  | **badly** | 41.75244^*^ | 3.27160 | <.001 | 32.5670 | 50.9379 |
| **rather well**  **N=2994** | **well** | -5.29899 | 2.01765 | .086 | -10.9638 | .3658 |
|  | **neither well nor badly** | 11.43378^*^ | 1.87812 | <.001 | 6.1607 | 16.7069 |
|  | **rather badly** | 25.67311^*^ | 2.04825 | <.001 | 19.9224 | 31.4239 |
|  | **badly** | 36.45345^*^ | 3.15438 | <.001 | 27.5971 | 45.3098 |
| **neither well nor badly**  **N=2658** | **well** | -16.73277^*^ | 2.06896 | <.001 | -22.5417 | -10.9239 |
|  | **rather well** | -11.43378^*^ | 1.87812 | <.001 | -16.7069 | -6.1607 |
|  | **rather badly** | 14.23932^*^ | 2.09881 | <.001 | 8.3466 | 20.1320 |
|  | **badly** | 25.01967^*^ | 3.18745 | <.001 | 16.0705 | 33.9689 |
| **rather badly**  **N=1958** | **well** | -30.97210^*^ | 2.22454 | <.001 | -37.2178 | -24.7264 |
|  | **rather well** | -25.67311^*^ | 2.04825 | <.001 | -31.4239 | -19.9224 |
|  | **neither well nor badly** | -14.23932^*^ | 2.09881 | <.001 | -20.1320 | -8.3466 |
|  | **badly** | 10.78035^*^ | 3.29056 | .011 | 1.5416 | 20.0191 |
| **badly**  **N=599** | **well** | -41.75244^*^ | 3.27160 | <.001 | -50.9379 | -32.5670 |
|  | **rather well** | -36.45345^*^ | 3.15438 | <.001 | -45.3098 | -27.5971 |
|  | **neither well nor badly** | -25.01967^*^ | 3.18745 | <.001 | -33.9689 | -16.0705 |
|  | **rather badly** | -10.78035^*^ | 3.29056 | .011 | -20.0191 | -1.5416 |

| **Table S.3 HSD estimation bias by Sleep Quality groups: Method-Self** vs. **Method-MCTQfree**.  The estimation bias between method Method-Self and Method-MCTQfree also became progressively larger with poorer sleep quality. The table presents post-hoc pairwise comparisons with Bonferroni corrections and shows that all five Sleep Quality groups were significantly different from each other, suggesting that underestimation of **HSD_self_** relative to **HSD_MCTQfree_** increases incrementally. * The mean difference is significant at the 0.05 level. | | | | | | |
| --- | --- | --- | --- | --- | --- | --- |
| **Sleep Quality groups** | **Sleep Quality groups** | **Estimation bias** | **Std. Error** | **Sig.** | **95% Confidence Interval** | |
|  |  |  |  |  | **Lower Bound** | Upper Bound |
| **well**  **N=2059** | **rather well** | 10.11416^*^ | 2.43713 | <.001 | 3.2716 | 16.9568 |
|  | **neither well nor badly** | 24.57254^*^ | 2.49911 | <.001 | 17.5559 | 31.5892 |
|  | **rather badly** | 38.91375^*^ | 2.68704 | <.001 | 31.3695 | 46.4580 |
|  | **badly** | 47.08818^*^ | 3.95179 | <.001 | 35.9930 | 58.1834 |
| **rather well**  **N=2994** | **well** | -10.11416^*^ | 2.43713 | <.001 | -16.9568 | -3.2716 |
|  | **neither well nor badly** | 14.45838^*^ | 2.26859 | <.001 | 8.0890 | 20.8278 |
|  | **rather badly** | 28.79959^*^ | 2.47410 | <.001 | 21.8532 | 35.7460 |
|  | **badly** | 36.97402^*^ | 3.81020 | <.001 | 26.2763 | 47.6717 |
| **neither well nor badly**  **N=2658** | **well** | -24.57254^*^ | 2.49911 | <.001 | -31.5892 | -17.5559 |
|  | **rather well** | -14.45838^*^ | 2.26859 | <.001 | -20.8278 | -8.0890 |
|  | **rather badly** | 14.34121^*^ | 2.53517 | <.001 | 7.2234 | 21.4591 |
|  | **badly** | 22.51564^*^ | 3.85014 | <.001 | 11.7058 | 33.3254 |
| **rather badly**  **N=1958** | **well** | -38.91375^*^ | 2.68704 | <.001 | -46.4580 | -31.3695 |
|  | **rather well** | -28.79959^*^ | 2.47410 | <.001 | -35.7460 | -21.8532 |
|  | **neither well nor badly** | -14.34121^*^ | 2.53517 | <.001 | -21.4591 | -7.2234 |
|  | **badly** | 8.17443 | 3.97470 | .397 | -2.9851 | 19.3340 |
| **badly**  **N=599** | **well** | -47.08818^*^ | 3.95179 | <.001 | -58.1834 | -35.9930 |
|  | **rather well** | -36.97402^*^ | 3.81020 | <.001 | -47.6717 | -26.2763 |
|  | **neither well nor badly** | -22.51564^*^ | 3.85014 | <.001 | -33.3254 | -11.7058 |
|  | **rather badly** | -8.17443 | 3.97470 | .397 | -19.3340 | 2.9851 |
|  | | | | | | |

| **Table S.4** **Statistics for the Bland-Altman plot presented in Fig. 4a in the main text: estimation bias and the agreement limits by Sleep Quality groups: Method-Self vs. Method-MCTQwork** | | | | | | | | |
| --- | --- | --- | --- | --- | --- | --- | --- | --- |
| **Sleep Quality groups** | | **HSD estimation bias**  Mean ± SD | **Standard error** | **t value** | **Limits of agreement** | | **Confidence**  **intervals** | |
| well | (N=2059) | -15.8 ± 62.8 | 1.4 | -11.4 | -138.8 | 107.2 | -18.5 | -13.1 |
| rather well | (N=2994) | -21.1 ± 63.0 | 1.2 | -18.3 | -144.6 | 102.4 | -23.4 | -18.9 |
| neither well nor badly | (N=2658) | -32.5 ± 69.5 | 1.3 | -24.1 | -168.8 | 103.7 | -35.2 | -29.9 |
| rather badly | (N=1958) | -46.8 ± 78.6 | 1.8 | -26.3 | -200.9 | 107.3 | -50.3 | -43.3 |
| badly | (N=599) | -57.6 ± 100.5 | 4.1 | -14.0 | -254.5 | 139.4 | -65.6 | -49.5 |
|  | | | | | | | | |
| **Table S.5** **Statistics for the Bland-Altman plot presented in Fig. 4b in the main text: estimation bias and the agreement limits by Sleep Quality groups: Method-Self vs. Method-MCTQfree** | | | | | | | | |
| **Sleep Quality groups** | | **HSD estimation bias**  **Mean ± SD** | **Standard error** | **t value** | **Limits of agreement** | | **Confidence**  **intervals** | |
| well | (N=2059) | -53.9 ± 76.8 | 1.7 | -31.8 | -204.5 | 96.7 | -57.2 | -50.6 |
| rather well | (N=2994) | -64.0 ± 78.9 | 1.4 | -44.4 | -218.6 | 90.5 | -66.9 | -44.4 |
| neither well nor badly | (N=2658) | -78.5 ± 83.7 | 1.6 | -48.3 | -242.6 | 85.7 | -81.7 | -75.3 |
| rather badly | (N=1958) | -92.8 ± 92.7 | 2.1 | -44.3 | -274.4 | 88.8 | -96.9 | -88.7 |
| badly | (N=599) | -101.0 ± 116.8 | 4.8 | -21.2 | -329.9 | 127.9 | -77.8 | -62.2 |

**Table S.6 Weighted least squares stepwise regressions with Sleep Quality, SJL, age, sex, BMI as predictors of HSDs**

Several multiple regressions were run to predict HSDs and the HSD estimation bias from Sleep Quality, SJL, sex, age and BMI.

This model explained 13.7% of the variance in **HSD_self_** (Adjusted R^2^(Sleep Quality) = 0.127)**;** stadardazied β (Sleep Quality) = -0.357), BMI was insignificant**.** In contrast, the model explained only 4.2% of the variance in **HSD_MCTQweek_** with Sleep Quality being a leading predictor (Adjusted R^2^ (Sleep Quality) = 0.021; β (Sleep Quality) = -0.147), BMI and SJL were insignificant. Only 3.6% of the variance in **HSD_MCTQwork_** were explained by the model, with Sleep Quality being a leading predictor (Adjusted R^2^ (Sleep Quality) = 0.021; β (Sleep Quality) = -0.149), BMI was insignificant. The model explained 10.8% of the variance in **HSD_MCTQfree_** with SJL being the leading predictor (Adjusted R^2^ (SJL) = 0.074; β (SJL) = 0.217), BMI was insignificant. Finally, the model explained 6.9% of the variance in **HSD estimation bias**, with Sleep Quality being a leading predictor (Adjusted R^2^ (Sleep Quality) = 0.062; β (Sleep Quality) = -0.244), age and BMI were insignificant.

| **Model HSD_self_** | | | | | | | | | |  |  |
| --- | --- | --- | --- | --- | --- | --- | --- | --- | --- | --- | --- |
| Model  **F(1, 10076) =404, p < 0.001** | R | R Square | Adjusted R Square | Std. Error of the Estimate | Change Statistics | | | | |  |  |
|  |  |  |  |  | R Square Change | F Change | df1 | df2 | Sig. F Change | St.Beta |  |
| **Sleep Quality** | 0.356 | 0.127 | 0.127 | 68.9781 | 0.127 | 1475.077 | 1 | 10135 | <.001 | -0.357 |  |
| Age | 0.366 | 0.134 | 0.134 | 68.6955 | 0.007 | 84.559 | 1 | 10134 | <.001 | -0.103 |  |
| Sex | 0.369 | 0.136 | 0.136 | 68.6291 | 0.002 | 20.619 | 1 | 10133 | <.001 | 0.042 |  |
| SJL | 0.371 | 0.138 | **0.137** | 68.57316 | 0.001 | 17.540 | 1 | 10132 | <.001 | -0.042 |  |
| *BMI - excluded | | | | | | | | | |  |  |
|  |  |  |  |  |  |  |  |  |  |  |  |
| **Model HSD_MCTQweek_** | | | | | | | | | |  |  |
| Model  **F(1, 10136) =147, p < 0.001** | R | R Square | Adjusted R Square | Std. Error of the Estimate | Change Statistics | | | | |  |  |
|  |  |  |  |  | R Square Change | F Change | df1 | df2 | Sig. F Change | St.Beta |  |
| **Sleep Quality** | 0.144 | 0.021 | 0.021 | 70.37772 | 0.021 | 215.578 | 1 | 10135 | <.001 | -0.149 |  |
| Age | 0.176 | 0.031 | 0.031 | 70.01804 | 0.01 | 105.394 | 1 | 10134 | <.001 | -0.105 |  |
| Sex | 0.205 | 0.042 | **0.042** | 69.62143 | 0.011 | 116.788 | 1 | 10133 | <.001 | 0.105 |  |
| *SJL, BMI - excluded | | | | | | | | | |  |  |
|  |  |  |  |  |  |  |  |  |  |  |  |
| **Model HSD_MCTQwork_** | | | | | | | | | |  |  |
| Model  **F(1, 10136) =96, p < 0.001** | R | R Square | Adjusted R Square | Std. Error of the Estimate | Change Statistics | | | | |  |  |
|  |  |  |  |  | R Square Change | F Change | df1 | df2 | Sig. F Change | St.Beta |  |
| **Sleep Quality** | 0.146 | 0.021 | 0.021 | 75.2514 | 0.021 | 220.161 | 1 | 10135 | <.001 | -0.149 |  |
| Sex | 0.173 | 0.030 | 0.030 | 74.92097 | 0.009 | 90.593 | 1 | 10134 | <.001 | 0.097 |  |
| SJL | 0.181 | 0.033 | 0.033 | 74.81417 | 0.003 | 29.955 | 1 | 10133 | <.001 | -0.081 |  |
| Age | 0.192 | 0.037 | **0.036** | 74.66029 | 0.004 | 42.813 | 1 | 10132 | <.001 | -0.069 |  |
| *BMI - excluded | | | | | | | | | |  |  |
|  |  |  |  |  |  |  |  |  |  |  |  |
|  |  |  |  |  |  |  |  |  |  |  |  |
| **Model HSD_MCTQfree_** | | | | | | | | | |  |  |
| Model  **F(1, 10136) =307, p < .001** | R | R Square | Adjusted R Square | Std. Error of the Estimate | Change Statistics | | | | |  |  |
|  |  |  |  |  | R Square Change | F Change | df1 | df2 | Sig. F Change | St.Beta |  |
| **SJL** | 0.271 | 0.074 | 0.074 | 80.85098 | 0.074 | 806.089 | 1 | 10135 | <.001 | 0.217 |  |
| Age | 0.299 | 0.090 | 0.089 | 80.15487 | 0.016 | 177.802 | 1 | 10134 | <.001 | -0.142 |  |
| Sleep Quality | 0.316 | 0.100 | 0.099 | 79.7178 | 0.01 | 112.426 | 1 | 10133 | <.001 | -0.104 |  |
| Sex | 0.329 | 0.108 | **0.108** | 79.34373 | 0.009 | 96.772 | 1 | 10132 | <.001 | 0.093 |  |
| *BMI - excluded | | | | | | | | | |  |  |
|  |  |  |  |  |  |  |  |  |  |  |  |
| **Model HSD estimation bias** | | | | | | | | | |  |  |
| Model  **F(1, 10136) =252, p < 0.001** | R | R Square | Adjusted R Square | Std. Error of the Estimate | Change Statistics | | | | |  |  |
|  |  |  |  |  | R Square Change | F Change | df1 | df2 | Sig. F Change | St.Beta |  |
| **Sleep Quality** | 0.248 | 0.062 | 0.062 | 62.64733 | 0.062 | 665.307 | 1 | 10135 | <.001 | -0.244 |  |
| Sex | 0.257 | 0.066 | 0.066 | 62.50049 | 0.004 | 48.678 | 1 | 10134 | <.001 | -0.067 |  |
| SJL | 0.264 | 0.069 | **0.069** | 62.39069 | 0.003 | 36.702 | 1 | 10133 | <.001 | -0.058 |  |
| *Age, BMI - excluded | | | | | | | | | |  |  |

**Table S.7 Weighted least squares stepwise regressions with ISI score, SJL, age, sex, BMI as predictors of HSDs.**

Sleep Quality strongly correlated with ISI continuous score (ρS = 0.70, p<0.001) and was also significantly predicted by it (simple regression Adjusted R^2^ = 0.525, F(1, 10203) = 11255.4, p<0.001). Several multiple regressions were run to predict HSDs and the HSD estimation bias from ISI, SJL, sex, age and BMI. In the last model additional predictor, the Sleep Quality, was added.

| **Model HSDself** | | | | | | | | | | |  | |
| --- | --- | --- | --- | --- | --- | --- | --- | --- | --- | --- | --- | --- |
| Model | R | R Square | Adjusted R Square | Std. Error of the Estimate | Change Statistics | | | | |  | |  |
|  |  |  |  |  | R Square Change | F Change | df1 | df2 | Sig. F Change | St.Beta | |  |
| **ISI** | 0.289 | 0.084 | 0.084 | 70.54744 | 0.084 | 920.399 | 1 | 10075 | <.001 | -0.297 | |  |
| Age | 0.307 | 0.094 | 0.094 | 70.14486 | 0.011 | 116.979 | 1 | 10074 | <.001 | -0.118 | |  |
| Sex | 0.311 | 0.097 | 0.096 | 70.05347 | 0.002 | 27.299 | 1 | 10073 | <.001 | 0.05 | |  |
| SJL | 0.312 | 0.098 | **0.097** | 70.02088 | 0.001 | 10.38 | 1 | 10072 | 0.001 | -0.033 | |  |
| *BMI -excluded | | |  |  |  |  |  |  |  |  | |  |
|  |  |  |  |  |  |  |  |  |  |  | |  |
| **Model HSD_MCTQweek_** | | | | | | | | | | |  | |
| Model | R | R Square | Adjusted R Square | Std. Error of the Estimate | Change Statistics | | | | |  | |  |
|  |  |  |  |  | R Square Change | F Change | df1 | df2 | Sig. F Change | St.Beta | |  |
| **ISI** | 0.118 | 0.014 | 0.014 | 70.63888 | 0.014 | 141.39 | 1 | 10075 | <.001 | -0.132 | |  |
| Age | 0.159 | 0.025 | 0.025 | 70.227 | 0.012 | 119.528 | 1 | 10074 | <.001 | -0.113 | |  |
| Sex | 0.192 | 0.037 | **0.037** | 69.81831 | 0.011 | 119.282 | 1 | 10073 | <.001 | 0.107 | |  |
| *SJL, BMI – excluded | | |  |  |  |  |  |  |  |  | |  |
| **Model HSD_MCTQwork_** | | | | | | | | | | |  | |
| Model | R | R Square | Adjusted R Square | Std. Error of the Estimate | Change Statistics | | | | |  | |  |
|  |  |  |  |  | R Square Change | F Change | df1 | df2 | Sig. F Change | St.Beta | |  |
| **ISI** | 0.124 | 0.015 | 0.015 | 75.44396 | 0.015 | 157.321 | 1 | 10075 | <.001 | -0.131 | |  |
| Sex | 0.156 | 0.024 | 0.024 | 75.10548 | 0.009 | 92.014 | 1 | 10074 | <.001 | 0.098 | |  |
| SJL | 0.162 | 0.026 | 0.026 | 75.02802 | 0.002 | 21.814 | 1 | 10073 | <.001 | -0.075 | |  |
| Age | 0.176 | 0.031 | **0.031** | 74.85144 | 0.005 | 48.58 | 1 | 10072 | <.001 | -0.074 | |  |
| *BMI – excluded | |  |  |  |  |  |  |  |  |  | |  |
| **Model HSD estimation bias** | | | | | | | | | | |  | |
| Model | R | R Square | Adjusted R Square | Std. Error of the Estimate | Change Statistics | | | | |  | |  |
|  |  |  |  |  | R Square Change | F Change | df1 | df2 | Sig. F Change | St.Beta | |  |
| **ISI** | 0.201 | 0.04 | 0.040 | 63.28036 | 0.040 | 422.246 | 1 | 10075 | <.001 | -0.193 | |  |
| Sex | 0.210 | 0.044 | 0.044 | 63.16067 | 0.004 | 39.221 | 1 | 10074 | <.001 | -0.061 | |  |
| SJL | 0.215 | 0.046 | **0.046** | 63.08153 | 0.002 | 26.29 | 1 | 10073 | <.001 | -0.050 | |  |

*Age, BMI – excluded


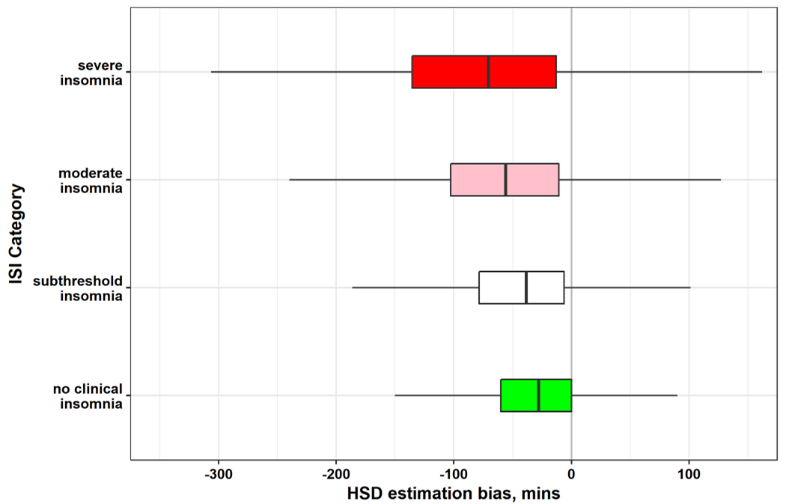
**Fig. S.1 Boxplots of HSD estimation bias by ISI category**.

| **Model HSD estimation bias with both Sleep Quality and ISI** | | | | | | | | | | |  | |
| --- | --- | --- | --- | --- | --- | --- | --- | --- | --- | --- | --- | --- |
| Model | R | R Square | Adjusted R Square | Std. Error of the Estimate | Change Statistics | | | | | |  | |
|  |  |  |  |  | R Square Change | F Change | df1 | df2 | Sig. F Change | St.Beta | |  |
| **Sleep Quality** | 0.247 | 0.061 | 0.061 | 62.59435 | 0.061 | 653.599 | 1 | 10075 | <.001 | -0.219 | |  |
| Sex | 0.255 | 0.065 | 0.065 | 62.4562 | 0.004 | 45.618 | 1 | 10074 | <.001 | -0.063 | |  |
| SJL | 0.262 | 0.069 | 0.068 | 62.34309 | 0.003 | 37.589 | 1 | 10073 | <.001 | -0.057 | |  |
| ISI | 0.263 | 0.069 | 0.069 | 62.32922 | 0.001 | 5.485 | 1 | 10072 | 0.019 | -0.033 | |  |

*Age, BMI – excluded

**Table S.8** **Sensitivity analysis in the long COVID-19 subgroup: HSD estimation bias and agreement limits between Method-Self and Method-MCTQweek**

To assess whether the HSD estimation bias has a different presentation in a long COVID-19 subgroup, we performed the main analyses (as in sections 3.1 and 3.2) . This group included 934 participants who had COVID-19 infection and met the WHO criteria for long COVID-19. The mean age of this group was 43.31 ± 14.04 years old (vs. 43.16 ± 16.81 in the general sample), see additional socio-demographic details in Table 1, main text. The distributions of **HSD_self_, HSD_MCTQweek_** and **HSD estimation bias** are shown in the **Figure SM-2**. The mean bias was -47.60. ± 72.42 minutes, a few minutes larger than in the general sample (-42.41 ± 67.42 minutes). The level of agreement between the two HSD assessment methods was within ±142 minutes, like the main sample**.** In contrast to the main sample, Pearson test demonstrated a significant slope of the regression line (k=0.17, Beta = 0.19, p < 0.001), suggesting that participants with symptoms of long COVID-19 have a tendency to underestimate their **HSD_self_** more in the low end (shorter sleep durations) as compared to the high end (longer sleep durations) of the HSD values range. The one-way ANOVA analysis showed that the magnitude of the estimation bias was dependent on Sleep Quality (F(4, 929)=6.47, p < 0.001). Altogether, this sensitivity analysis confirmed that the participants of the long COVID-19 subgroup had HSD estimation bias similar to the general sample.


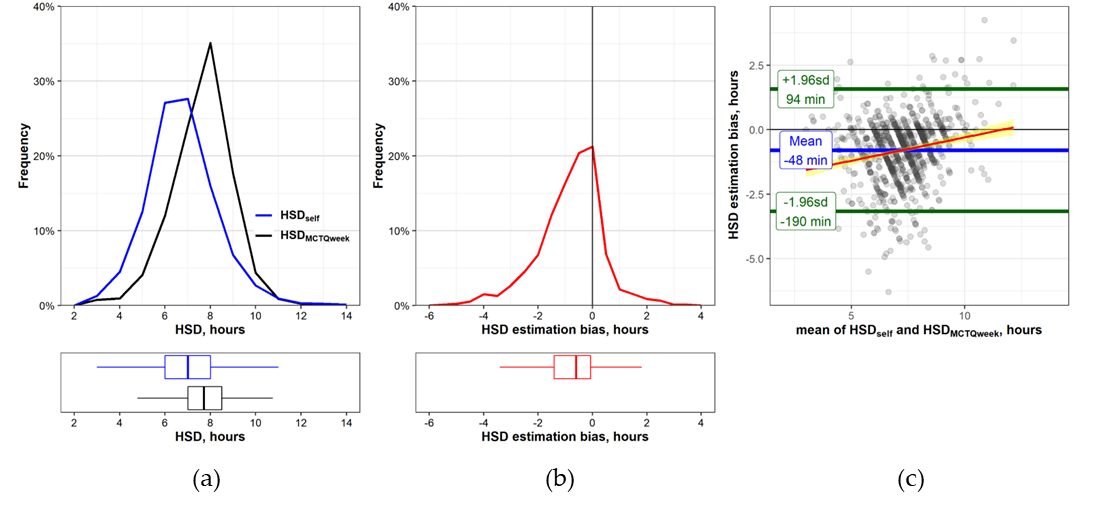
**Fig. S.2 Long COVID-19 subgroup data (N=934). Habitual sleep duration (HSD) by Method-Self and Method-MCTQweek.** (**a**) Upper panel - HSD distribution, percent from group total by method: blue line - **HSD_self_**, black line - **HSD_MCTQweek._** Lower panel - Boxplots of individual HSD by method. Whiskers - max and min values, box borders – 75th and 25th percentiles, line through the box – median. (**b**) Upper panel - HSD estimation bias values distribution, percent from group total. Lower panel - Boxplot of individual HSD estimation bias values. (**c**) **Bland-Altman plot comparing Method-Self and Method-MCTQweek.** The blue line indicates that the Method-Self sleep duration estimates are on average 48 minutes shorter than Method-MCTQ estimates. The green lines indicate the 95% limits of agreement (±1.96SDs). The linear regression line (red) shows that the HSD estimation bias is decreasing (reaches zero values) as the mean of **HSD_self_** and **HSD_MCTQweek_** range values increase.

**Table S. 9** **Sensitivity analysis in the elderly (>65y) subgroup: HSD estimation bias and agreement limits between Method-Self and Method-MCTQweek**

To assess whether the HSD estimation bias has a different presentation in a the elderly (>65y) subgroup, we performed the main analyses (as in sections 3.1 and 3.2) . This group included 1187 participants. The mean age of this group was 71.22 ± 3.68 years old (vs. 43.16 ± 16.81 in the general sample), see additional socio-demographic details in Table 1, main text. The distributions of **HSD_self_, HSD_MCTQweek_** and **HSD estimation bias** are shown in the **Figure SM-3**. The mean bias was -44.17 ± 42 minutes, two minutes larger than in the general sample (-42.41 ± 67.42 minutes). The level of agreement between the two HSD assessment methods was within ± 126 minutes, also similar to the main sample**.** The one-way ANOVA analysis showed that the magnitude of the estimation bias was dependent on Sleep Quality (F(4, 1057)=5.69, p < 0.001), as in the general sample. Altogether, this sensitivity analysis confirmed that the participants of the elderly subgroup had HSD estimation bias similar to the general sample.

**
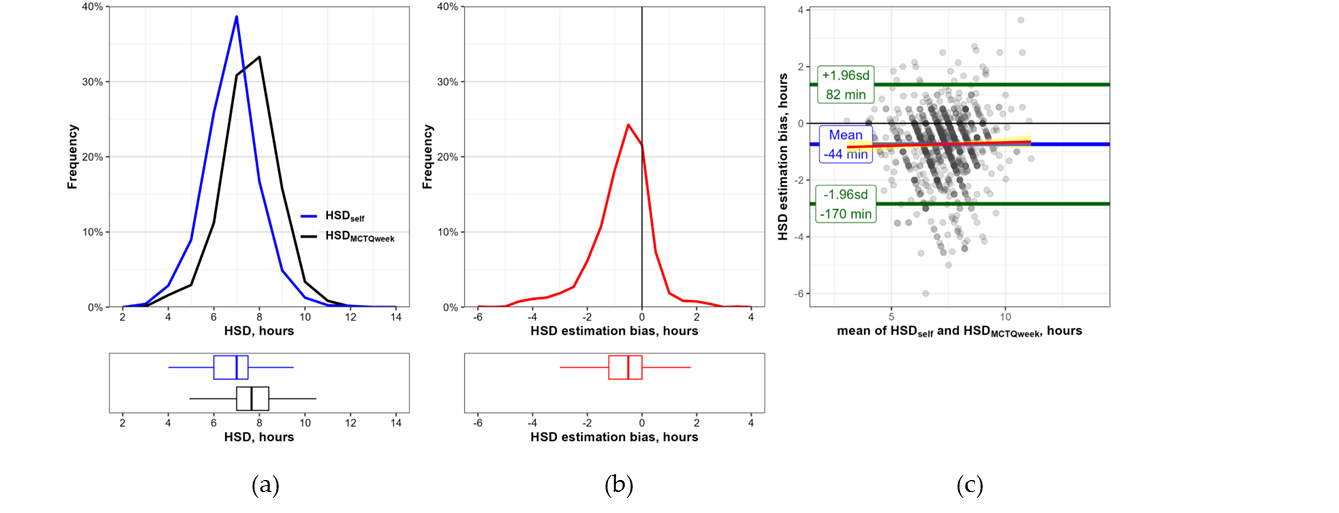
**

**Fig. S.3 Elderly (>65) subgroup data (N=1187). Habitual sleep duration (HSD) by Method-Self and Method-MCTQweek.** (**a**) Upper panel - HSD distribution, percent from group total by method: blue line - **HSD_self_**, black line - **HSD_MCTQweek._** Lower panel - Boxplots of individual HSD by method. Whiskers - max and min values, box borders – 75th and 25th percentiles, line through the box – median. (**b**) Upper panel - HSD estimation bias values distribution, percent from group total. Lower panel - Boxplot of individual HSD estimation bias values. (**c**) **Bland-Altman plot comparing Method-Self and Method-MCTQweek.** The blue line indicates that the Method-Self sleep duration estimates are on average 48 minutes shorter than Method-MCTQ estimates. Pearson test demonstrated that the slope of the regression line was insignificant (red line). The green lines indicate the 95% limits of agreement (±1.96SDs).

**Table S.8. Ethical approval data.**

| **Country** | **Ethical diary number** | **Notes** |
| --- | --- | --- |
| Austria | NA | Due to the anonymous nature of survey collection, the Ethical Board of the Medical University of Vienna, Austria did not require an ethical evaluation or approval. |
| Brazil | NA | Due to the anonymous nature of survey collection, the Regional Ethical Board did not require an ethical evaluation or approval. |
| Bulgaria | Protocol Number 46/05.08.2021 | Ethics Commission of the Institute of Neurobiology, Bulgarian Academy of Sciences |
| Canada | 2020-151-A-1-R-1 21-05-2021  REB#20-5540 | The “Comité d’éthique de l’Université Laval » reviewed and approved this research protocol.  University Health Network Research Ethics Board, Toronto, Ontario, Canada |
| China (Hong Kong) | 2020.277 | Joint Chinese University of Hong Kong-New Territories East Cluster Clinical Research Ethics Committee |
| Croatia | 100-21/21-4 (07.05.2021) | Ethics Committee of the Institute for Medical Research and Occupational Health |
| Finland | NA | Due to the anonymous nature of survey collection, the Regional Ethical Board did not require an ethical evaluation or approval. |
| France | NA | Declaration jas been made to the CNIL (Comité National Informatique et liberté).  Due to the anonymous nature of survey collection, the Ethical Board did not require an ethical evaluation or approval. |
| Germany | EA1/162/20. | Ethics Committee of Charite University Hospital Berlin |
| Israel | AU-HEA-MK-20210603 | The study was approved by the Ariel University Human Research Ethics Committee of the Faculty of Health Sciences |
| Italy | protocol number: 0000861, April 24, 2021 | Institutional Ethics Committee of the Department of Psychology of the Sapienza University of Rome |
| Japan | No. 198/2020 | The ethics committee of the Neuropsychiatric Research Institute, Tokyo, Japan |
| Norway | NA | Due to the anonymous nature of survey collection, the Regional Ethical Board did not require an ethical evaluation or approval. |
| Portugal | CES-UCP nº142, May 27th, 2022 | The project was approved unanimously by the Life Sciences Ethical Commission of the Portuguese Catholic University. |
| Sweden | NA | Due to the anonymous nature of survey collection, the Regional Ethical Board did not require an ethical evaluation or approval. |
| USA | IRB-20-257 | Deemed exempt by the Mississippi State University Institutional Review Board. |

NA = non-applicable.
